# Shear Elastic Modulus on Patellar Tendon Captured from Supersonic Shear Imaging: Correlation with Tangent Traction Modulus Computed from Material Testing System and Test–Retest Reliability

**DOI:** 10.1371/journal.pone.0068216

**Published:** 2013-06-27

**Authors:** Zhi Jie Zhang, Siu Ngor Fu

**Affiliations:** 1 Department of Rehabilitation Sciences, the Hong Kong Polytechnic University, Hung Hom, Kowloon, Hong Kong; 2 Department of Physiotherapy, Guangdong Provincial Work Injury Rehabilitation Hospital, Guangzhou, China; The University of Queensland, Australia

## Abstract

Characterization of the elastic properties of a tendon could enhance the diagnosis and treatment of tendon injuries. The purpose of this study was to examine the correlation between the shear elastic modulus on the patellar tendon captured from a Supersonic Shear Imaging (SSI) and the tangent traction modulus computed from a Material testing system (MTS) on 8 fresh patellar pig tendons (Experiment I). Test–retest reliability of the shear elastic modulus captured from the SSI was established in Experiment II on 22 patellar tendons of 11 healthy human subjects using the SSI. Spearman Correlation coefficients for the shear elastic modulus and tangent traction modulus ranged from 0.82 to 1.00 (all *p*<0.05) on the 8 tendons. The intra and inter-operator reliabilities were 0.98 (95% CI: 0.93–0.99) and 0.97 (95% CI: 0.93–0.98) respectively. The results from this study demonstrate that the shear elastic modulus of the patellar tendon measured by the SSI is related to the tangent traction modulus quantified by the MTS. The SSI shows good intra and inter-operator repeatability. Therefore, the present study shows that SSI can be used to assess elastic properties of a tendon.

## Introduction

Tendons are involved in every human motion and subjected to high loads. A tendon consists of parallel collagen fibers to resist elongation [1] and exhibits viscoelastic properties for force production and absorption [2]. Alteration in tendon stiffness may compromise the tendon’s capacity to absorb and respond to loads [3,4]. Quantification of its elastic properties may help improve our understanding of the underlying causes of tendon-related disorders, such as tendinopathy.

The elastic properties of tendons have been determined using animal [5] and cadaveric [6] tendons undergoing ramped stretching imposed by a motor of a material testing system (MTS). It has not yet been established, however, whether findings from isolated excised tendons can be applied to *in-vivo* physiological functions [4]. Ultrasonography is a non-invasive method for measuring the elastic properties of the human tendon *in-vivo* [4,7]. This method has been used to examine changes in tendon stiffness associated with exercise [8] and aging [9]. However, complex methodologies and long acquisition time are the drawbacks of this approach [7].

Recently, ultrasound elastography has been applied to investigate the mechanical properties of the Achilles tendon [10]. Ultrasound elastography (strain imaging) is a real-time imaging tool for the in vivo estimation of tissue strain distribution [11,12]. A compressive force is applied to the tissue surface inducing transverse tissue displacement, which is calculated from the echo signal set before and after the compression [13]. The force can be applied manually (freehand elastography) or mechanically (transient elastography). The absolute value of the elastic properties cannot be provided from freehand ultrasound elastography. Manual compression may alter the mechanical properties of the testing tissues.

Supersonic shear imaging (SSI) operates on a transient elastography principle. It produces elastography images based on the combination of a radiation force and an ultrafast ultrasound acquisition imaging system capable of capturing in real time, the propagation of the resulting shear waves [14]. The elastic modulus can be calculated from the velocity of the propagating wave when a faster velocity indicates a greater elastic modulus. Therefore, the elastic modulus can be calculated by measuring the propagation of shear waves. A light touch on the skin with the ultrasound probe is suggested by the manufacturer and a quantitative elasticity map can be computed from the system within a few milliseconds. The objectives of this study were: (1) to assess the correlation of the shear elastic modulus captured from an SSI and the tangent traction modulus from a MTS (Experiment I); and (2) to assess the reliability of the shear elastic modulus captured from the SSI, by using test–retest measurements on the patellar tendons of healthy subjects (Experiment II).

## Experiment I

## Methods

**Figure 1:**
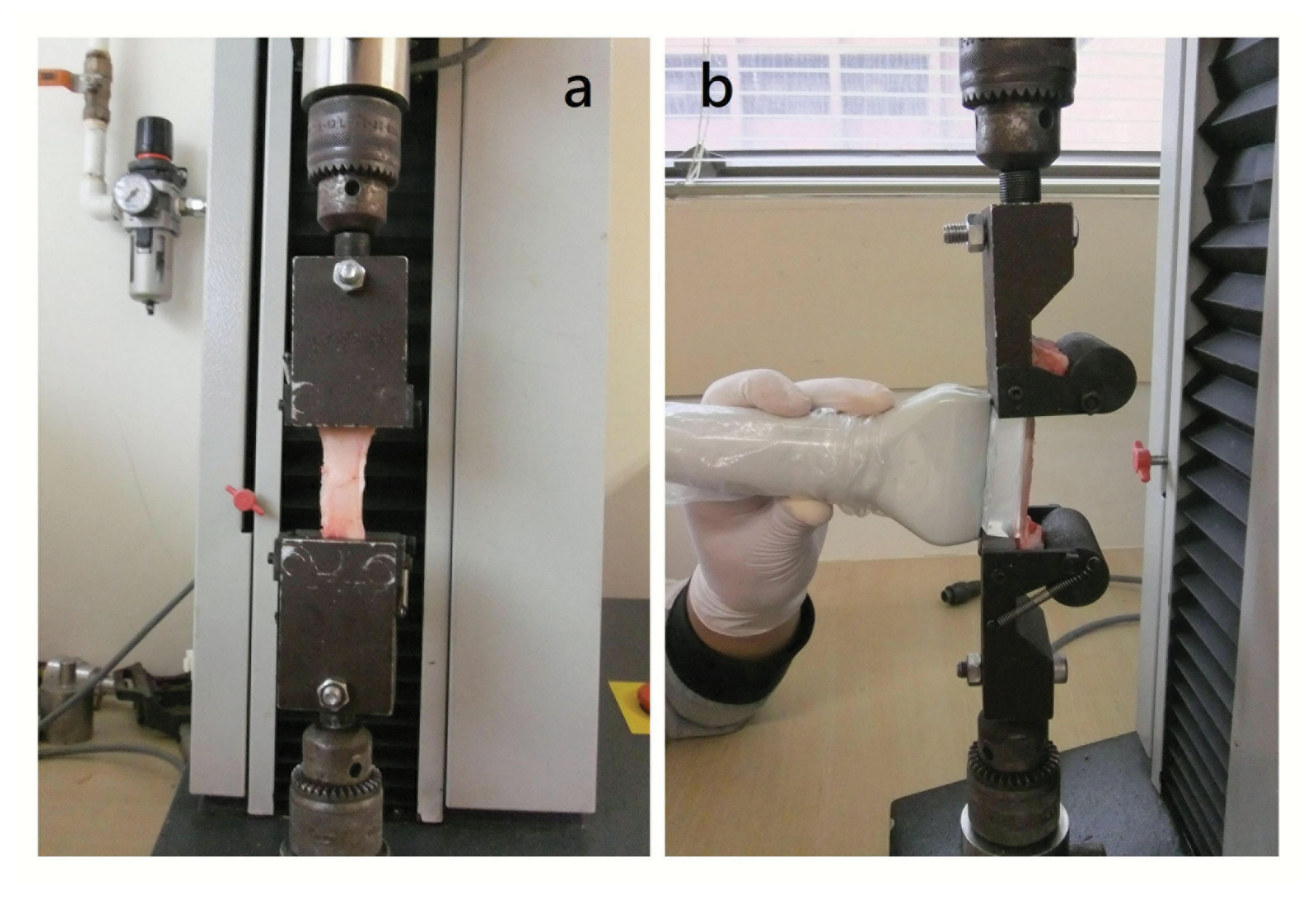
Experiment set-up. (**a**) The tendon was clamped carefully onto the MTS (anterior view); (**b**) The gel pad and transducer were put lightly on the tendon in order to capture its elasticity using Supersonic Shear Imaging.

**Figure 2:**
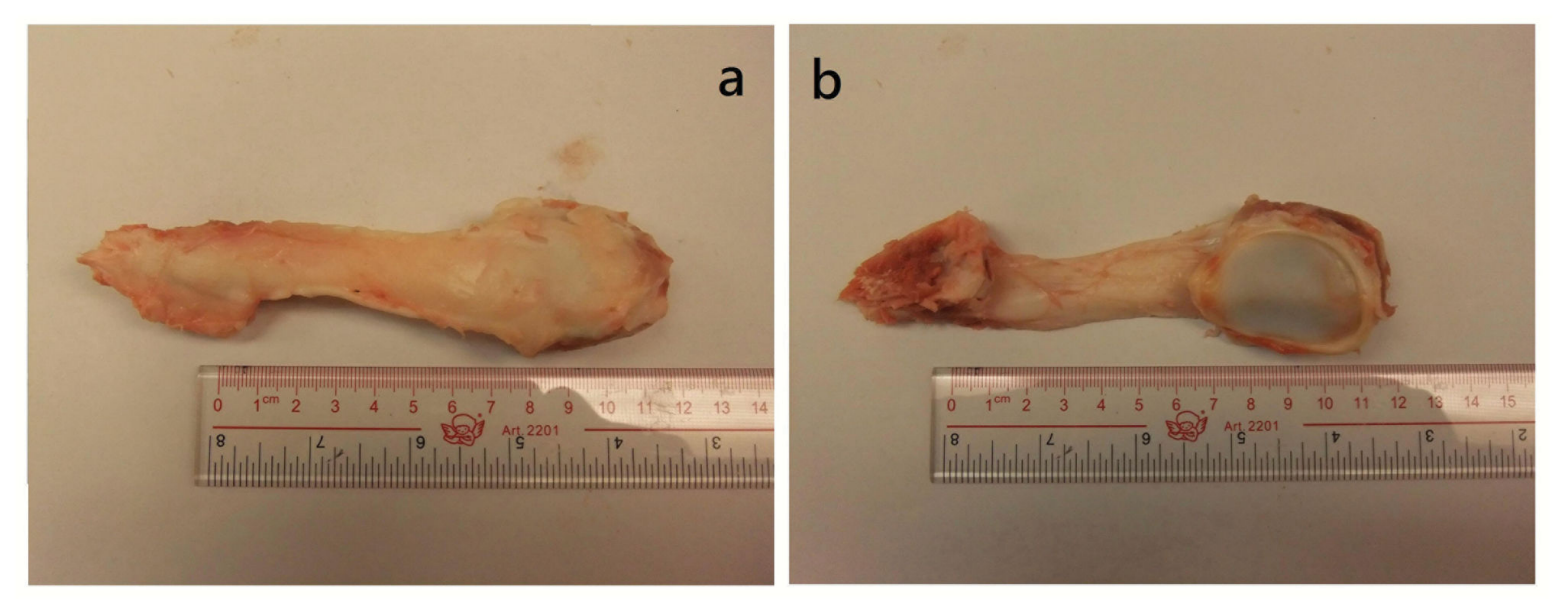
Photographs of a representative patellar tendon. (**a**) Anterior view for patellar tendon; (**b**) The length of the patellar tendon was measured by a meter.

Fresh knee joints of pigs are dissected and sold in local food market. It was not necessary, therefore, to apply for ethics approval. A total of eight fresh patellar pig tendons were dissected carefully from the patella and tibia, and all soft tissues were removed from around the knee joints, leaving only patella and small tibia tuberosity (Figure 1). The length of the specimens were measured by a plastic meter (Smartmax; SM-103) that was used to adjust the distance of the 2 clamps of a material testing system (MTS Synergie 200, MTS System Corporation, Ivry sur Seine Cedex, France) (Figure 2). The room temperature was controlled at 25 ^°^C.

The patella and tibia of the dissected patellar tendon were connected to the 2 clamps with their fibers aligned using applied force. The tensile force (F) was applied to the 2 crossheads causing incremental displacement of the 2 clamps (d) in steps of 0.2mm at a test speed of 20mm/min until the force reached 10N. A maximum force of 10N was chosen based on our own pilot study. When the applied force exceeded 10N, the elastic modulus reached the saturation level of SSI (300kPa). The force was captured by the load cells and the displacement of the crossheads was measured by an extensometer (MTS model 634.12F-24, MTS System Corporation, Eden Prairie, MN). Both values were displayed on-line on a computer attached to the material testing system.

After the specimen was secured between the two clamps, a gel pad (ULTRA PHONIC FOCUS; Confoming gel pad; USA) was fixed onto the surface of the specimen in order to capture clear imaging. The cross-sectional area of the tendon was measured using the B-mode of the An Aixplorer® ultrasound unit (Supersonic Imaging, Aix-en-Provence, France) in conjunction with a linear-array transducer at 4-15 MHz frequency and a high frame rate (up to 20,000 frames/s). Once a clear image of the tendon had been achieved, the shearwave elastography mode was activated. To avoid the effect of anisotropy on the measurement, the probe was aligned with the direction of the fibres. One image was captured from each increment of 0.2mm when the tension was constant during the elastographic measurements. The image was frozen when the entire ROI was covered by the color and stored for off-line analysis.

**Figure 3:**
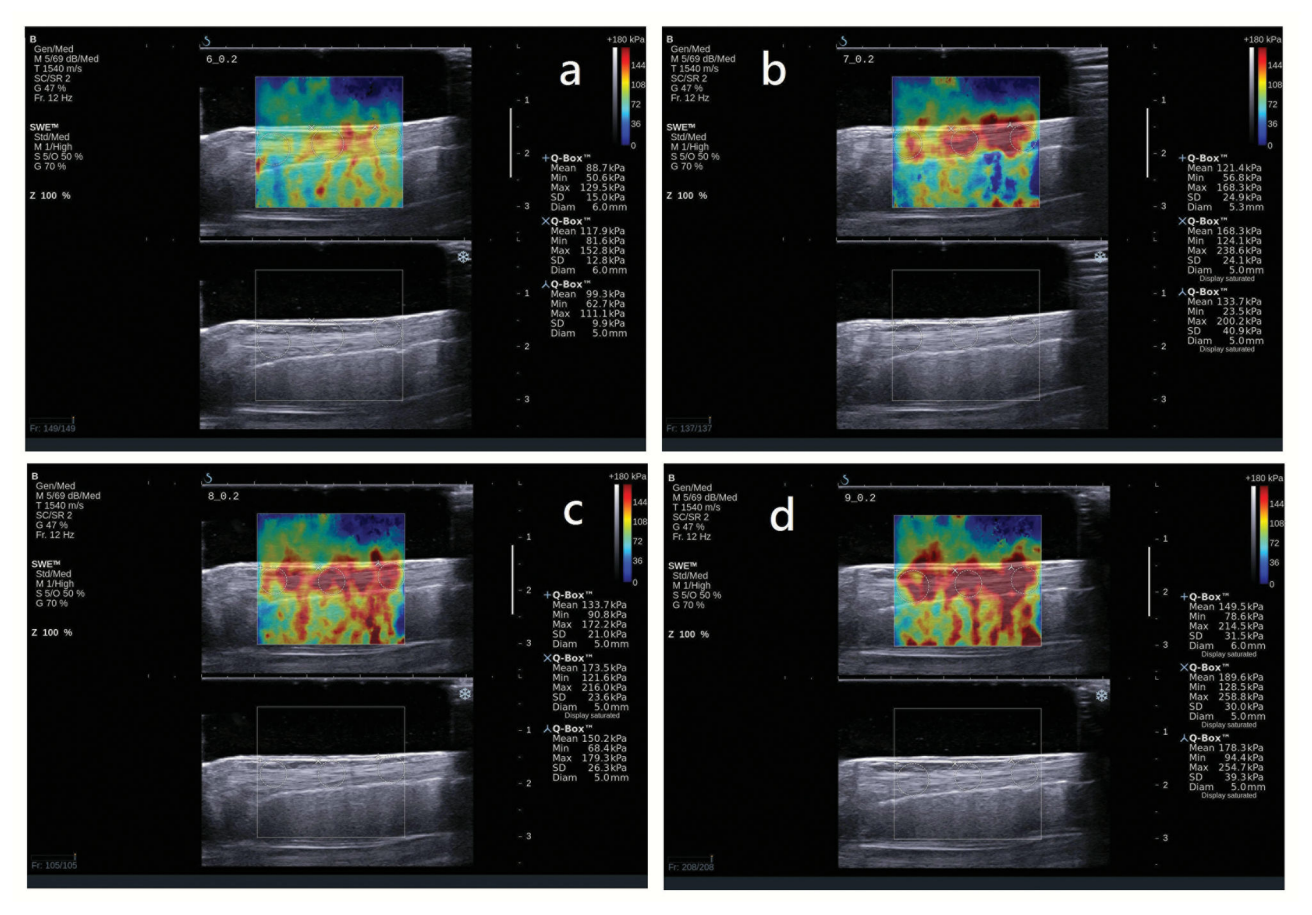
Changes of shear elastic modulus of pig patella tendon (PT) during increasing loading (from a to d). Upper images show the color-coded box presentation of the PT elasticity (red color represents stiffer area and green color represents softer area) with the measurement circle representing the region of interest and its corresponding shear elastic modulus demonstrating under Q-Box^TM^ on the right. The 3 measurement circles were placed on the proximal, middle and distal part of the PT (The diameter of the measurement circle was dependent on the thickness of the tendon). The bottom images show the longitudinal grey scale sonograms of the PT to ensure the capture of clear images.

Off-line analysis was conducted on the captured images from the SSI. A circle that delineated the region of interest (ROI) for the measurement of the elastic modulus was placed at the proximal, middle and distal parts of the SSI acquisition box on the patella tendon (Figure 3). The colours represented the stiffness of the tissues within the region of interest and ranged from red (hard) to blue (soft). The diameter of the ROI was determined by the width of the patellar tendon. Mean values of the elastic modulus on the patella tendon within the ROI were assessed by the built-in specific quantification program. The elastic modulus (E) was computed from the system based on the following equation, E = 3ρV_s_^2^. Where density ρ is assumed to be constant (1000kg/m^3^) in human soft tissue and V_s_ is the velocity of the shear wave propagation[14]. Due to tendon anisotropy, the shear elastic modulus was used in this study [15]. Thus, all the values obtained using the SSI was divided by 3 in this study.

Data processing was conducted using the software LabVIEW 8.6 (LabVIEW Professional Development System, USA). The loads (F) and crosshead displacement (d) were recorded during testing by a computer attached to the MTS. The tensile stress (σ) was calculated as the applied load divided by the tendon cross-sectional area (A_0_) (σ=F/A_0_). The tensile strain (ε) was calculated as displacement divided by initial length (L_0_) (ε=d/L_0_). The tangent modulus was computed by a self-written programme based on the formula ***E*= Δσ/Δε**. Spearmen’s rank correlation tests were used to assess the level of correlation between the shear elastic modulus of the tendon captured from the SSI system and the tangent traction modulus calculated from the MTS.

## Experiment II

The subjects were fully informed of the procedures as well as the purpose of this study. Written consent was obtained from each subject. This study protocol was approved by the Human Subject Ethics Subcommittee of the Department of Rehabilitation Science, the Hong Kong Polytechnic University.

Eleven healthy subjects (8 male, 3 female; age: 26.1+3.2 years, weight: 58.7+12.3 kg, height: 169.2+10.0cm) were invited to participate in this study. These subjects underwent a clinical examination to exclude patellar tendon disorders, such as patellar tendinopathy. A further exclusion criterion for participation of healthy subjects was a history of knee injury or surgery. Clinical examinations, consisting of an assessment of local tenderness over the patellar tendon and pain aggravation during single leg squatting were performed by an experienced physiotherapist.

Each participant was examined while lying supine with the knee at 30° of flexion [16]. The knee was supported on a firm towel and a custom-made ankle stabilizer was used to keep the leg in neutral alignment on the coronal and transverse planes. Prior to testing the subject was allowed to have 5 minutes rest in this position, to ensure the elastic modulus of the patellar tendon was evaluated at resting status. The room temperature was controlled at 25°C.

The elastic modulus of the patellar tendon was measured using an Aixplorer® ultrasound unit (Supersonic Imaging, Aix-en-Provence, France) in conjunction with a linear-array transducer at 4-15 MHz frequency and a high frame rate (up to 20,000 frames/s). The transducer was placed longitudinally on the patellar tendon with the knee flexion of 30°. The shear wave elastography mode was then activated to measure the elastic modulus of the proximal part of the patellar tendon. The transducer was stationed on the skin, with a light pressure on top of a generous amount of coupling gel, perpendicularly on the skin’s surface. The transducer was kept motionless for 8-12 seconds during the acquisition of the SSI sonogram [17]. Images were frozen when the color in the region of interest was uniform and were then stored for off-line analyses. In total, 3 images were captured for the tendon on each knee.

Two operators (I and II) participated in the inter-operator investigation. Operator I had about 5 years of experience in ultrasound scanning and SSI training. Operator II was a sports physiotherapist with about 2 years of experience in ultrasound imaging as well as SSI short course training. The operators took turns to examine each subject’s patellar tendon at one-hour intervals; and by Operator II with a 3-hour interval. The results were not communicated until all subjects had been examined.

**Figure 4:**
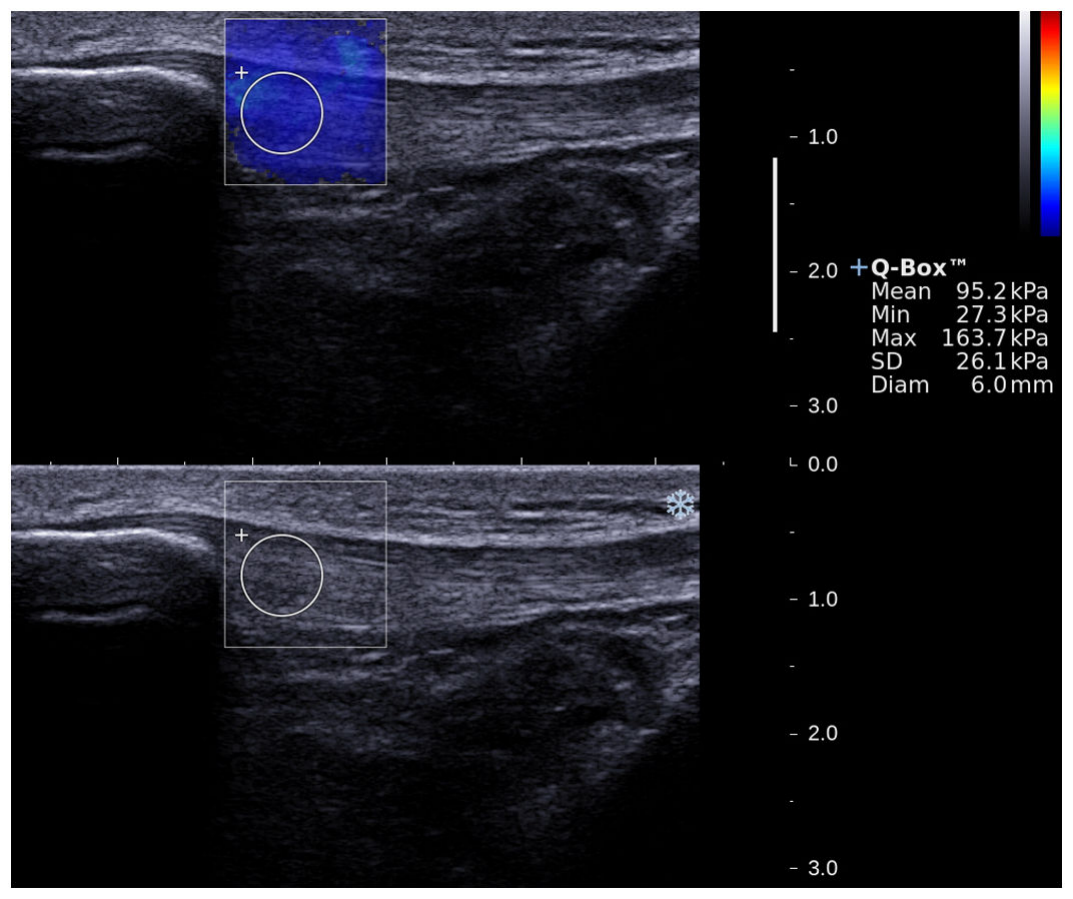
Typical example of elastic modulus measurement for the proximal patellar tendon on a healthy subject. Upper images show the color-coded box presentation of the PT elasticity (the red color represents the stiffer area and the green color represents the softer area) with the measurement circle representing the region of interest and its corresponding elastic modulus demonstrating under Q-Box^TM^ on the right. The transducer was kept motionless for 8 to 12 seconds during the acquisition of the SSI sonogram and the diameter of the measurement circle was defined by the thickness of the tendon. The bottom images show the longitudinal grey scale sonograms of the PT to ensure the capture of clear images.

A circle that delineated the region of interest (ROI) was centered at the proximal part of the tested tendon (Figure 4). The diameter of ROI was defined by the thickness of the tendon, which was the distance between the superior and inferior borders of the proximal part of the patellar tendon. The mean values of the elastic modulus on the patellar tendon within the ROI were computed from the system.

The dependent measure for analysis was the averaged mean tendon shear elastic modulus from all 3 images of the patellar tendon. Both intra and inter-operator reliability were examined using intraclass correlation coefficients (ICC). ICC(1,3) was used to determine the intra-operator reliability and ICC (2,2) was computed to examine the inter-operator reliability [18]. The coefficient of variance (CV) was calculated [using the formula CV = (standard deviation/mean) ×100%]. The standard error measurement was computed (using the formula SEM= standard deviation × √ 1-ICC), and minimal detectable difference was calculated (using the formulaMDD=1.96×SEM× √ 2). All reliability coefficients were interpreted as follows: below 0.499 as poor, 0.500 to 0.699 as moderate, 0.700 to 0.899 as good, and 0.900 to 1.000 as excellent [19]. The statistical analysis was performed using SPSS Version 17.0 for Windows (SPSS Inc, Chicago, IL).

## Results

### Experiment I

**Figure 5:**
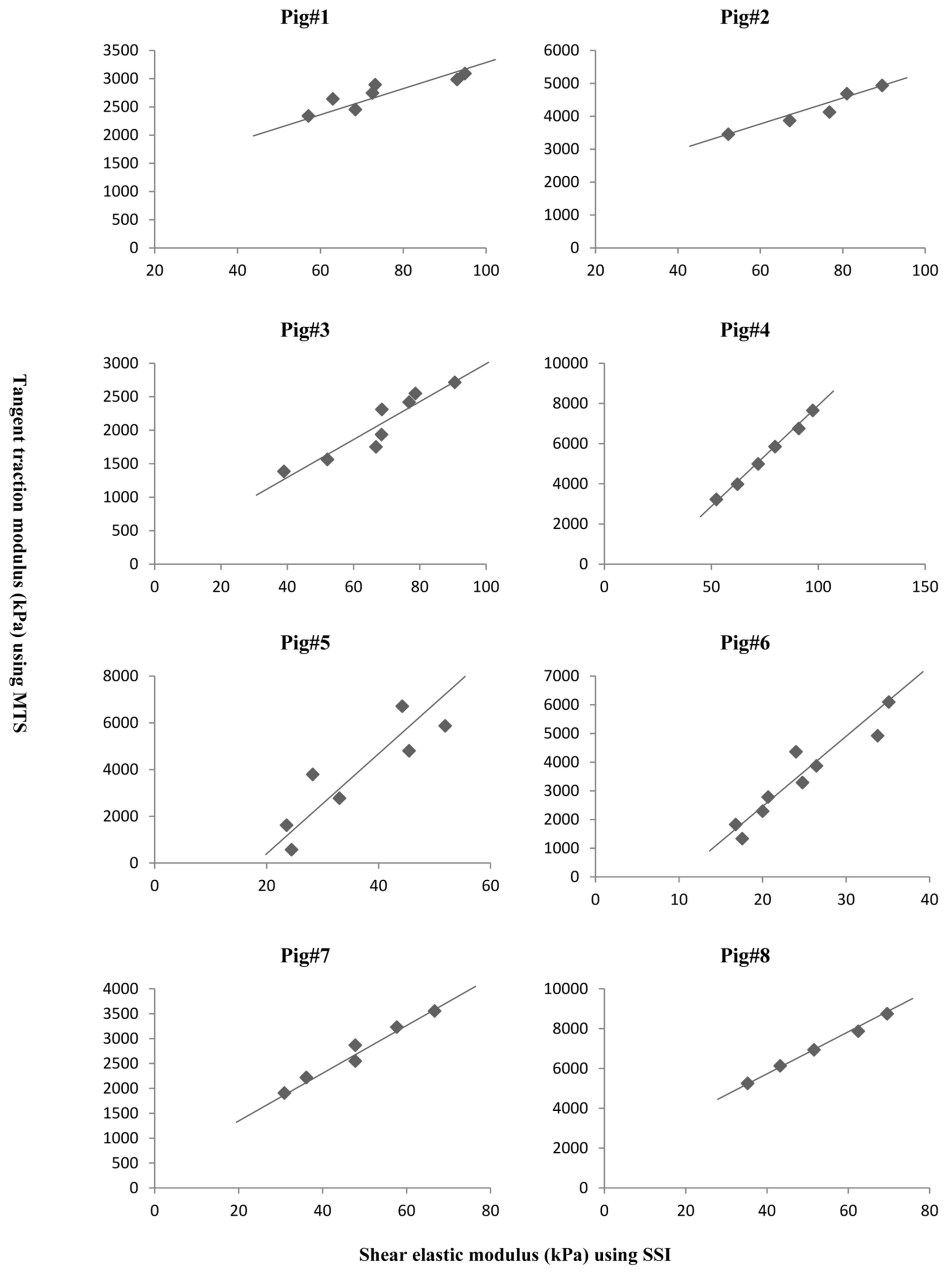
Correlations between shear elastic modulus of tendon captured from a Shear supersonic imaging and tangent traction modulus of tendon computed from a material testing system.

Table 1 shows the cross sectional area, resting length, shear elastic modulus and tangent traction modulus obtained from the 8 fresh pig patellar tendons. Figure 5 depicts the relationships among the SSI and MTS measurements in the specimens. Significant correlations were found between the 2 variables in all tested specimens with the correlation coefficient ranging from 0.82 to 1.00 (all *p*<0.05, Table 2 Figure 5). 

**Table 1:** Cross sectional area, resting length, shear elastic modulus and tangent traction modulus obtained from fresh patellar tendon.

**pig**	**Cross sectional area (mm^2^)**	**Resting length (mm)**	**Mean shear elastic modulus (kPa)**	**Mean tangent traction modulus (kPa)**
1	86.3	40.0	74.6	2736.5
2	114.0	45.0	73.3	4216.5
3	95.3	42.0	67.6	2079.8
4	71.0	65.0	75.8	5410.5
5	48.6	44.0	35.8	3736.5
6	54.0	55.0	24.3	3420.4
7	48.3	50.0	47.8	2722.7
8	46.6	40.0	52.4	6989.3

MTS and SSI denote Material Testing System and Shear Supersonic Imaging.

**Table 2:** Spearmen’s rank correlation coefficient between the shear elastic modulus and tangent traction modulus of tendon obtained from SSI and MTS, respectively.

**pig**	***Rho***	***p-value***
1	0.96	0.000
2	1.00	0.000
3	1.00	0.000
4	1.00	0.000
5	0.82	0.023
6	0.93	0.000
7	0.99	0.000
8	1.00	0.000

### Experiment II

The intra and inter-operator reliabilities for the measurements of the proximal patellar tendons in the eleven healthy subjects (22 patellar tendons) were excellent. The intra-operator reliability value for ICC was 0.98 and for MDD was 4.27kPa, corresponding to an SEM of 1.54kPa and CV of 29.53%. With regard to the inter-operator reliability, the ICC value was 0.97 and the MDD value was 13.68kPa, corresponding to an SEM of 1.46kPa and a CV of 25.89% (Table 3).

**Table 3:** Intra-class coefficient (ICC) values for intra and inter-operator reliabilities on the shear elastic modulus of patellar tendon.

**Intra-operator reliability** (Mean±SD) (kPa)					
Test 1	Test 2	*ICC*	*95%CI*	*SEM*	*MDD*
36.9±10.9	36.0±9.5	*0.98*	*0.93-0.99*	*1.54kPa*	*4.27kPa*
**Inter-operator reliability**(Mean±SD) (kPa)					
Operator I	Operator II	*ICC*	*95%CI*	*SEM*	*MMD*
36.9±10.9	36.7±9.5	*0.97*	*0.93-0.98*	*1.64kPa*	*4.54kPa*

Abbreviations: ICC = intra correlation coefficient; CI = confidence index; SEM = standard error measurement; MDD = minimal detectable difference

## Discussion

Significant correlations were found between the shear elastic modulus on the patellar tendon captured from the SSI and the tangent traction modulus computed from the MTS. Excellent intra and inter-operator reliabilities were obtained when the SSI was performed on the healthy patellar tendons.

High correlations were observed between the shear elastic modulus measurement using the SSI and the tangent traction modulus obtained from the MTS of the 8 pig patellar tendons. We also observed differences in the slopes of different specimens. Hence, there was no single transformation from the elastic modulus to the tangent modulus that covered all specimens. We postulate that such differences might relate to the following factors. The MTS is known to have several inherent limitations. The stretching of the clamped fibrous tissues is associated with some slippage of inner fibres [4]. In this study, the bones (patella/ tibia tuberosity) were fixed tightly on the MTS. Some of the stretching force imposed from the MTS might have been taken up at the system-bone interface, which might have been different on each tendon. Soft tissues (fat/fascia) around the tendons had to be removed by hand. Any remaining tissues would have contributed to some of the loads. Despite great care being exercised to clear the tissue around the tendons, the amount of tissue remaining might have been different on each tendon. The tangent modulus computed from the MTS might thereby have been influenced by the above two technical issues to different extents on the tested tendons. On the other hand, intrinsic factors such as age and gender can contribute to the viscoelastic properties of the tendon. The viscoelastic properties of the patellar tendon may alter structurally with age, for example changes in collagen content. In an animal model, Haut et al. [20] reported a decrease in the collagen content of the patellar tendon with age. Similar observations were also found in human studies [21,22]. Gender may also affect the viscoelastic properties of the patellar tendon. Kubo [23] reported that the stiffness of the medial gastrocnemius tendon was significantly higher in males (approximately 37%) than in females. Note that, in this study, the elastic modulus was calculated based on the assumption that the density of the medium was a constant. This, however, may have been affected by the age or gender of the testing tendons, as we did not control for either of these factors.

To the best of our knowledge, the present investigation is the first to report the relationship between the modulus elastic using the SSI and the tangent traction modulus from the MTS measurement on tendons. All of the reported studies compared the muscle elastic modulus with muscle activities based on electromyography (EMG) studies. Nordez’s [24] was the first group to report that the elastic modulus of the biceps muscles of 6 healthy subjects was related strongly to the EMG activity level during muscle isometric contraction. Significant linear relationships between the elastic modulus and the individual muscle force were also reported on the small hand muscles such as the abductor digiti minimi and first dorsal interosseous [25], indicating that the elastic modulus could be used to estimate tension inside tissues. In addition, Maïsetti [27] reported a significant correlation between the elastic modulus and medial gastrocnemius muscle force during passive stretching. The correlations reported in the present study were possible only because both moduli were increased when the tension was increased.

The ultrasonography method has been used extensively over the last 20 years to assess the elastic properties of tendons. The elastic properties of the tibialis anterior and gastrocnemius tendons have been measured by ultrasound imaging as a method of estimating tendon-aponeurosis elongation during the tensile loading induced by the contraction of the in-series muscle [4]. Hansen [7] reported that the method to assess the elastic properties of human patellar tendon is reliable using ultrasonography with EMG during quadriceps muscle isometric contraction. The EMG has been used simply by some authors to correct force measurements from the antagonist contribution. Although this is a useful method to evaluate the elastic properties of the tendon, there are some limitations, such as complex techniques (transducer fixation technique), knee joint movement control, time consumption, complicated data analysis procedures and dependence on the muscle contraction [7]. Due to above the barriers, it is difficult to apply this method in the hospital and clinic to estimate the elastic properties of tendons. The SSI has overcome these limitations in the elastic properties measurement of the tendon, and this study has supported it as a relatively convenient method of measuring the elastic property of the patellar tendon.

Experiment II evaluated the intra and inter-operator reliability in obtaining the SSI elastic modulus measurements of the healthy participants’ proximal patellar tendons at the rest position within-day. If subjects with tendon disorders are assessed several times by different examiners, it is important to know the intra and inter-operator reliabilities. The results of this study have demonstrated that the SSI of the proximal patellar tendon has excellent intra and inter-operator reliabilities in healthy subjects. The SSI can be used to assess disease progression and the efficacy of intervention on patellar tendons when evaluations have to be conducted at different time points.

It has been reported that the SSI is a reliable method for evaluating the elastic properties of muscles. Lacourpaille [26] reported that the ICC value of intra- and inter-operator reliability among various muscles (gastrocnemius medialis/tibialis anterior/rectus femoris/biceps brachii/ triceps brachii/ vastus lateralis) during resting status ranged from 0.81 to 0.95 and 0.42 to 0.94, respectively. Another study revealed that the intra-operator reliability of the elastic properties of biceps brachii during 3% and 7% maximal EMG activity were good (3%, ICC=0.89; 7%, ICC=0.94) [23]. Our intra and inter-reliabilities results of 0.98 and 0.97 respectively are higher than those reported from these studies of muscles. One of the reasons for this may be related to the different structures of the muscle and tendon. A tendon consists of parallel collagen fibers, and it is easier to align and re-align the US probe with the tendon than with the muscle fibers, resulting in higher reliability.

Drakonaki et al. [10] obtained moderate to good intra and inter-operator reliability in assessing the stiffness of Achilles tendons using real-time freehand ultrasound elastography which depends on compressive force. In their study, 25 healthy subjects were recruited for the assessment of tendon stiffness at the middle third of the free tendon and the middle part between the myotendinous junction and the calcaneal insertion. The intra and inter-operator reliabilities ranged from moderate to good (0.51-0.78). One of the major differences between the SSI and real-time freehand ultrasound elastography is that the mechanical vibration is induced automatically by using a radiation force of ultrasound beams [14]. Thus, the SSI technique does not depend on external force from operators. This may be one of the main explanations why the SSI technique is more reliable than real-time free-hand ultrasound elastography.

In addition, our study calculated the MDD, which can provide a value to reflect a real change as a reference for future study. In terms of our findings, the measurement of the elastic modulus of the patellar tendon should be greater than 4.27kPa (the same operator) and 4.54kPa (different operator) to reflect real changes with retested measurements.

There are some advantages of the SSI technique when compared with other methods to evaluate the tendon elastic properties. First, it is a reliable and convenient technique to assess the elastic properties of the tendon. In the present study, the time required for scanning 2 tendons lasted for 5-8 minutes. Second, the operation of the machine can be learnt by a novice. Although Operator I (2years) and Operator II (5years) had different lengths of experience with the ultrasound scanning technique, the findings from the present study have demonstrated good intra and inter-reliabilities of the SSI measurement on the tendon elastic modulus, which indicates that the results could not have been influenced by the operator’s experience. Finally, the SSI can be used to evaluate tendon elastic properties that are not affected by the presence of pain. The conventional approach based on ultrasonography and the EMG, induces an increase in the tensile force on the tested tendon that might cause pain on a tendon with pathologies. These advantages of the SSI make it a promising clinical tool to follow disease progression and enhance the efficacy of different interventions.

In this study, the mean shear elastic modulus on the healthy patellar tendons ranged from 36.0 to 36.9kPa. The results from the present study were higher than those reported by Kot [17]. The mean shear elastic modulus on healthy patellar tendons being reported were 23 to 24 kPa. Such discrepancies might relate to the different method of defining the ROI. In Kot’s study, the ROI was pre-determined (2mm, 3mm or 4mm). In the present study, we adopted the approach used by Nordez and Hug [24]. The diameter of the ROI was defined by the thickness of the tendon, which was the distance between the superior and inferior borders of the proximal part of the patellar tendon. Note that the tendon thicknesses in this study ranged from 3 to 7mm and different portions of the patellar tendon contain different percentages of collagen fibers [28]. Our approach, thereby, included the whole rather than portion of the tendon.

## Conclusion

The present study has indicated that the shear elastic modulus on the patellar tendon measured from the SSI is related closely to the tangent traction modulus calculated from the MTS. The *in-vivo* measurement has illustrated excellent reliability of this tool. The SSI can be applied to evaluate the elastic properties of a healthy patellar tendon. The diagnostic role of this technique will be investigated by assessing the shear elastic modulus of normal and pathological tendons, as well as to monitor disease progression and the efficacy of intervention on individuals with tendon disorders.
